# *Tropheryma whipplei* Intestinal Colonization in Migrant Children, Greece

**DOI:** 10.3201/eid2809.220068

**Published:** 2022-09

**Authors:** Sofia Makka, Ioanna Papadogiannaki, Androniki Voulgari-Kokota, Theano Georgakopoulou, Myrto Koutantou, Emmanouil Angelakis

**Affiliations:** Hellenic Pasteur Institute, Athens, Greece (S. Makka, I. Papadogiannaki, A. Voulgari-Kokota, M. Koutantou, E. Angelakis);; National Public Health Organization, Marousi, Greece (T. Georgakopoulou)

**Keywords:** *Tropheryma whipplei*, Greece, migrants, children, bacteria, enteric infections

## Abstract

We obtained fecal samples from migrant children <12 years of age throughout hotspots in Greece and tested them for *Tropheryma whipplei* by using a quantitative PCR assay. We identified 6 genotypes of *T. whipplei*, 4 of which are newly described. Our findings suggest a high prevalence of *T. whipplei* in these regions.

*Tropheryma whipplei* is an intracellular bacterium recognized as the causative agent of enteric infection Whipple disease (*1*). *T. whipplei* intestinal colonization prevalence in humans depends on geographic area, age, and method of exposure (*2*). In Europe, *T. whipplei* has been detected in stool specimens in 2%–11% of healthy persons (*1*). The prevalence of *T. whipplei* intestinal colonization has been reported to be higher in children than in adults, suggesting an age-dependent presence (*3*). In developing countries, the rates are especially high, probably because of poor sanitary conditions (*3*). The prevalence of *T. whipplei* was shown to be high among children in Senegal (West Africa), reaching 75%; in contrast, the prevalence rate for children in France was 15% (*1*,*4*). *T. whipplei* has been associated with diarrhea in young children, suggesting a causative link between the bacterium and that symptom (*5*). Because migrants often live without resources that enable appropriate personal hygiene, and because they have limited access to healthcare, they are exposed to many communicable infections. As a result, migrant populations have a poorer standard of overall physical health compared with the general population, and they suffer from a disproportionate burden of communicable diseases, including those caused by parasites, enteroviruses, and *Mycobacterium tuberculosis* (*6*). In Greece, in collaboration with the Hellenic National Public Health Organization, we routinely test stool samples of persons classified as migrants, with the intent of determining the presence of *T. whipplei* and identifying genotypes circulating among migrant children.

We obtained stool specimens from children 0–12 years of age living in different hotspots throughout Greece and tested them for *T. whipplei*. We screened all samples by using a quantitative PCR that targeted 155-bp and 150-bp repeated sequences (*4*). For positive samples, we performed genotyping by using a multispacer system that targeted 4 highly variable genomic sequences (HVGSs) as previously described (*4*). The 4 HVGSs obtained from each specimen were compared with those available in the GenBank database and those listed on the Institut Hospitalo-Universitaire Méditerranée Infection website (Multispacer Typing—*T. whipplei*, https://ifr48.timone.univ-mrs.fr/mst/tropheryma_whipplei) to determine the corresponding genotype. We added all of the HVGSs we discovered to the Institut Hospitalo-Universitaire Méditerranée Infection website and performed Student *t* or χ^2^ tests by using Epi Info 6.0 (https://www.cdc.gov/epiinfo); we considered differences with a p value <0.05 to be significant.

We tested 128 stool samples obtained from 20 hotspots throughout Greece and identified 35 (27%) samples positive for *T. whipplei*. We found positive samples in 13 (65%) of the 20 hotspots we investigated, and we noted the highest presence of *T. whipplei* in the Alexandria hotspot, where all samples were positive, followed by the hotspot of Leros (Figure, panel A). The study population was 53% boys, and median age (+SD) was 5 (+3.8) years); the median age (+SD) of children whose stool samples tested positive was 4 (+3.6) years, and 19 (54%) of them were boys. Stool samples from boys tended to have increased bacterial loads (p =0.06), and stool samples from children 0–4 years of age had significantly higher bacterial loads (74%) compared with samples from children 5–12 years of age (33%) (p = 0.004).

**Figure:**
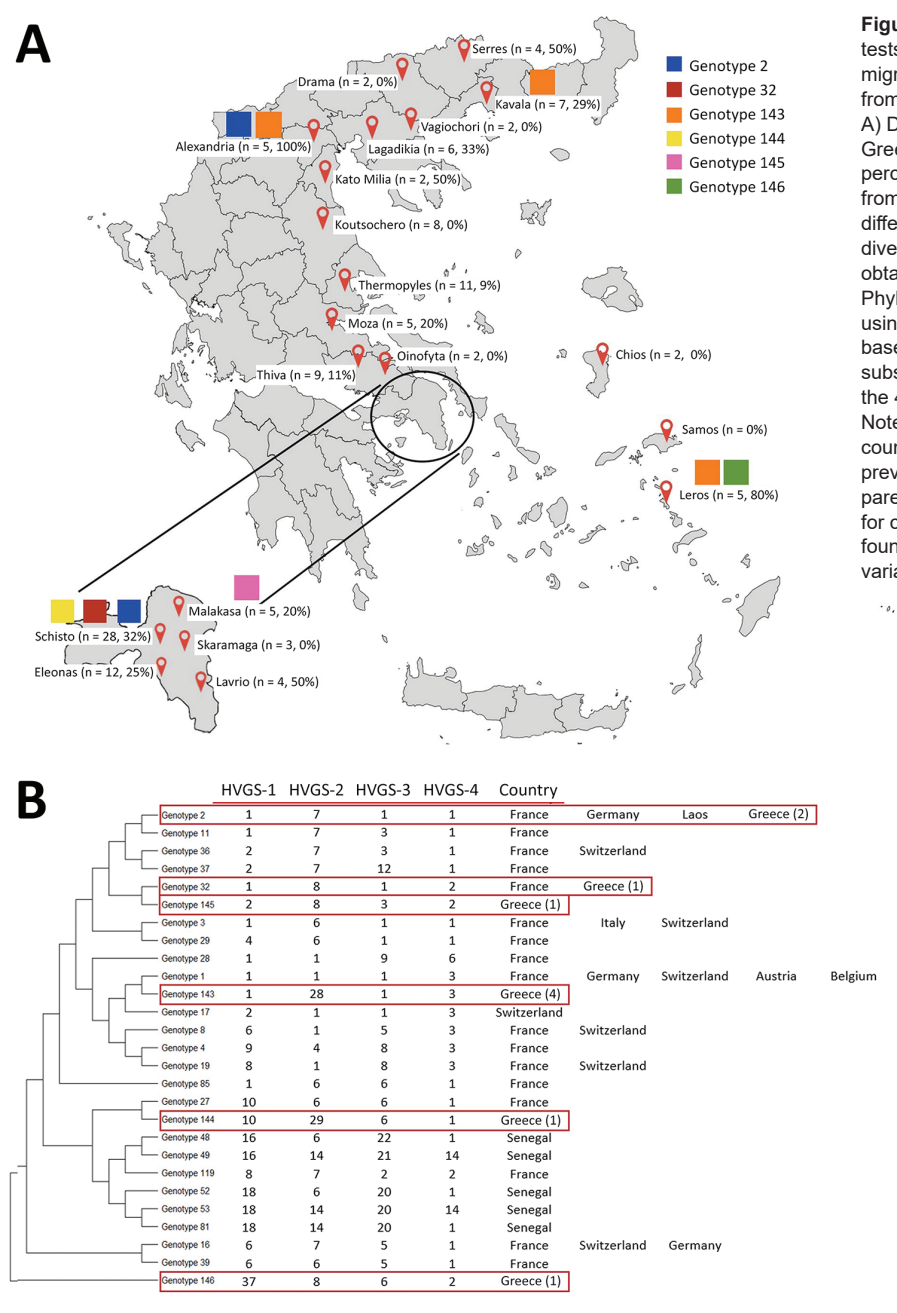
Results of stool sample tests for *Tropheryma whipplei* from migrant children 0–12 years of age from 20 hotspots throughout Greece. A) Defined hotspots throughout Greece, showing numbers and percentages of *T. whipplei* recovered from each location and distribution of different genotypes. B) Phylogenetic diversity of 6 genotypes of *T. whipplei* obtained from migrants (red boxes). Phylogenetic tree was constructed by using the maximum-likelihood method based on the Tamura 3-parameter substitution model. Sequences from the 4 HVGSs were concatenated. Noted next to the genotypes are the countries in which they have been previously detected. Numbers in parentheses note positive test results for children based on each genotype found in Greece. HVGS, highly variable genomic sequence.

Because of insufficient DNA loads, we obtained genotypes by reverse transcription PCR for only 10 of the 35 samples that tested positive for *T. whipplei*. We classified *T. whipplei* strains into 6 unique HVGS genotypes (Figure, panel B); most of them belonged to genotype 143, identified in 3 hotspots, followed by genotype 2, observed in 2 hostspots. We found 2 children from the same family in the Kavala hotspot who exhibited the same genotype, 143 (*7*). Our quantitative PCR has been previously evaluated (*8*), as has the HVGSs we used for genotyping (*4*).

Our identification of a high percentage of *T. whipplei* in stool samples from migrant children living in different hotspots throughout Greece supports prior data showing that persons living under poor hygienic conditions, particularly children, have increased rates of *T. whipplei* infection compared with the general adult population (*3*). A high percentage of *T. whipplei* infection was observed also in Ghana (*9*), and an upward tendency of *T. whipplei* was noted in Laos, Gabon, and Senegal (*3*,*9*).

We identified 6 genotypes of *T. whipplei*, including 4 newly described genotypes, in fecal samples from migrant children in Greece. The presence of genotype 143 in 3 hotspots suggests that this clone is possibly epidemic, and our results support the highly contagious nature of *T. whipplei*. To date, no specific genotypes have been associated with disease versus asymptomatic carriage, and the same genotype can be observed in acute infections, chronic infections, and asymptomatic carriage (*3*). The fact that 2 children from the same family exhibited the same genotype supports the hypothesis that *T. whipplei* can be transmitted between humans through saliva or feces, depending on hygiene conditions (*7*,*10*).

In conclusion, we provide evidence of a high prevalence of *T. whipplei* in migrant children throughout Greece. Because *T. whipplei* is associated with acute diarrhea in children (*5*), we emphasize the need for systematic surveillance in tracking this bacterium in immigrant populations.
